# Supplementary figures and images for: The assessment of acute chorioretinal changes due to intensive physical exercise in young adults
Source: PLoS One. 2022 May 25;17(5):e0268770. doi: 10.1371/journal.pone.0268770 (PMC9132279; doi:10.1371/journal.pone.0268770)

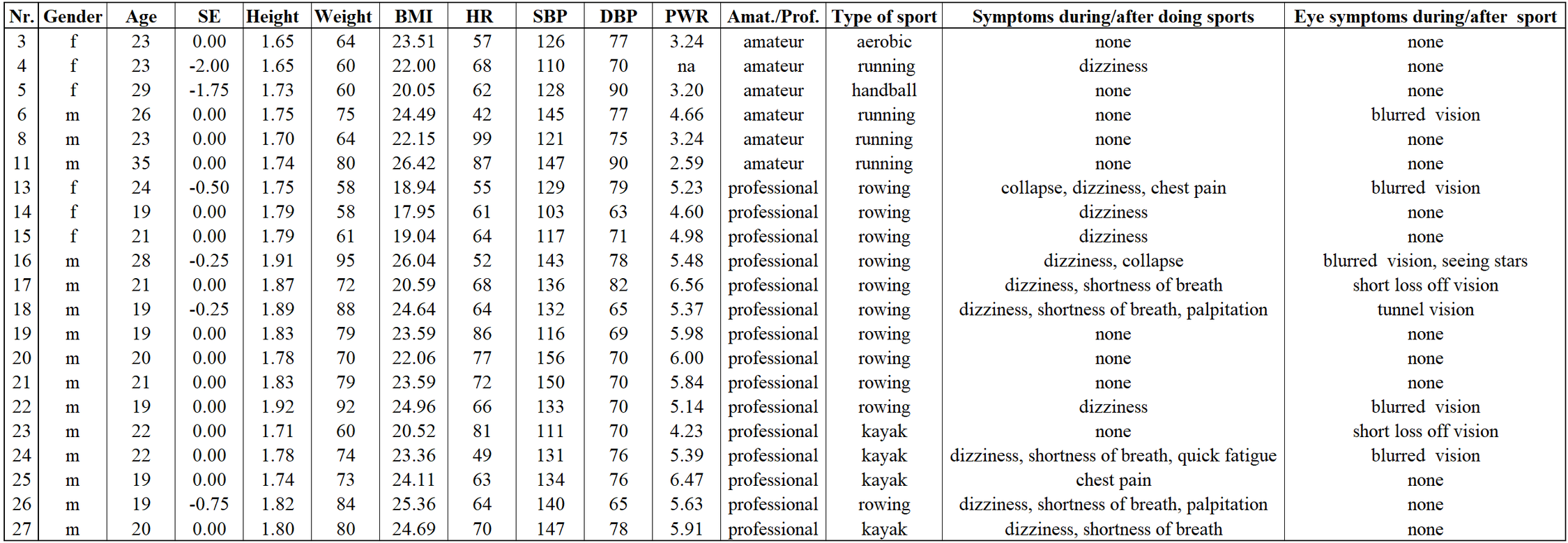

Supplement: S1 Table — Amateur vs. professional status, type of sports, systemic and ocular symptoms experienced during and after sports activity are highlighted. Abbreviations: body mass index (BMI), heart rate (HR), systolic and diastolic blood pressure (SBP and DBP, respectively), power to weight ratio (PWR, in the case of Subject 4 the data were corrupted during processing). (TIF) [file pone.0268770.s001.tif]

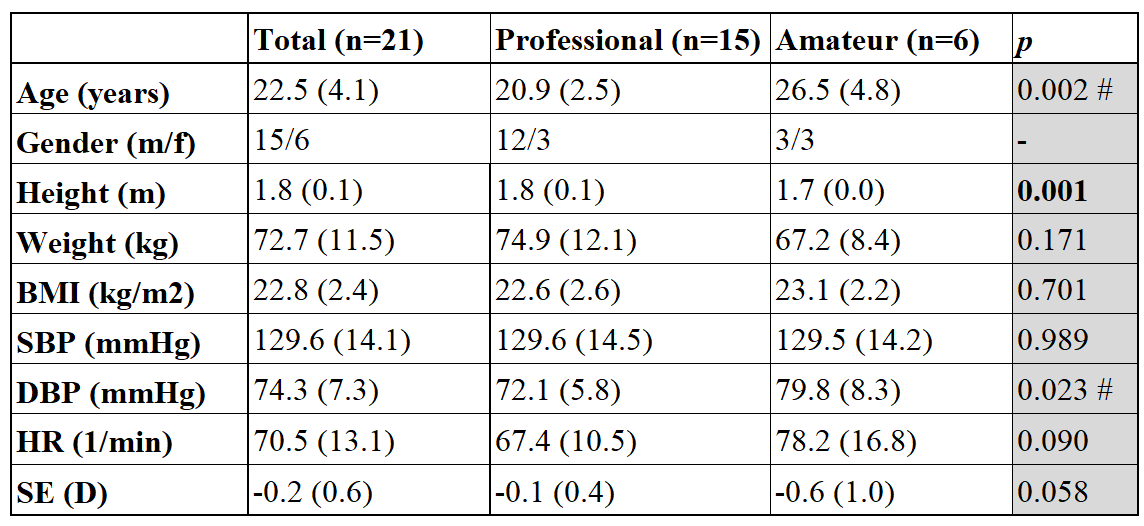

Supplement: S2 Table — Data are presented as means (SD), the p value refers to the comparison by Student’s t-test between professional (n = 15) and amateur sportsmen (n = 6). Significant data are highlighted in bold, # denotes missed significant results (with p values between 0.001 and 0.05). (TIF) [file pone.0268770.s002.tif]

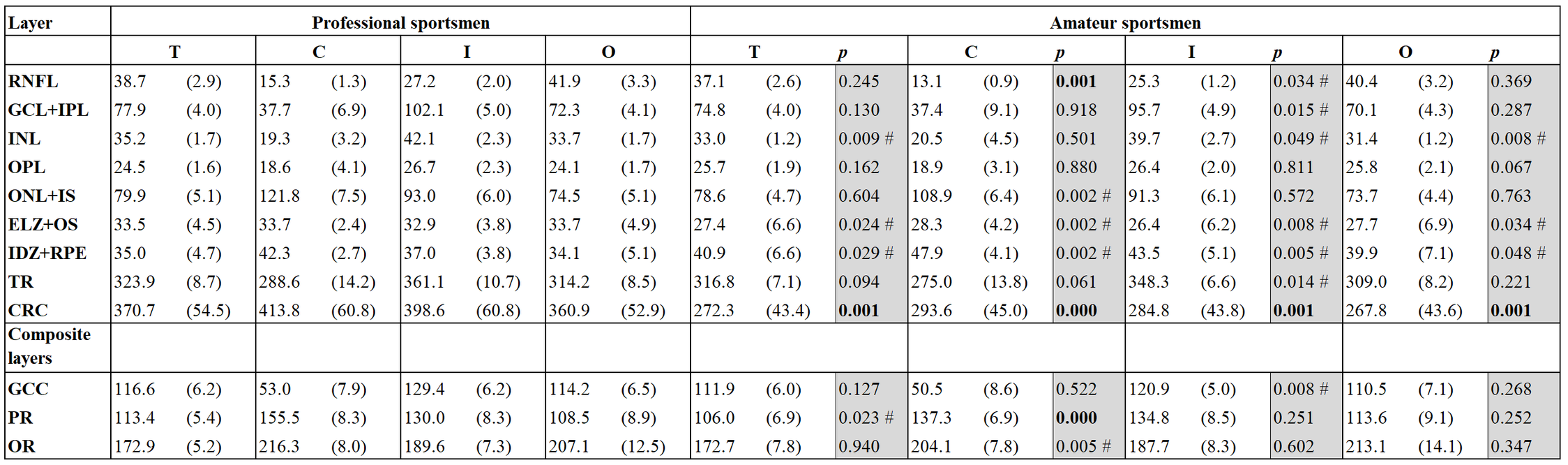

Supplement: S3 Table — For the abbreviations see Fig 2. (TIF) [file pone.0268770.s003.tif]

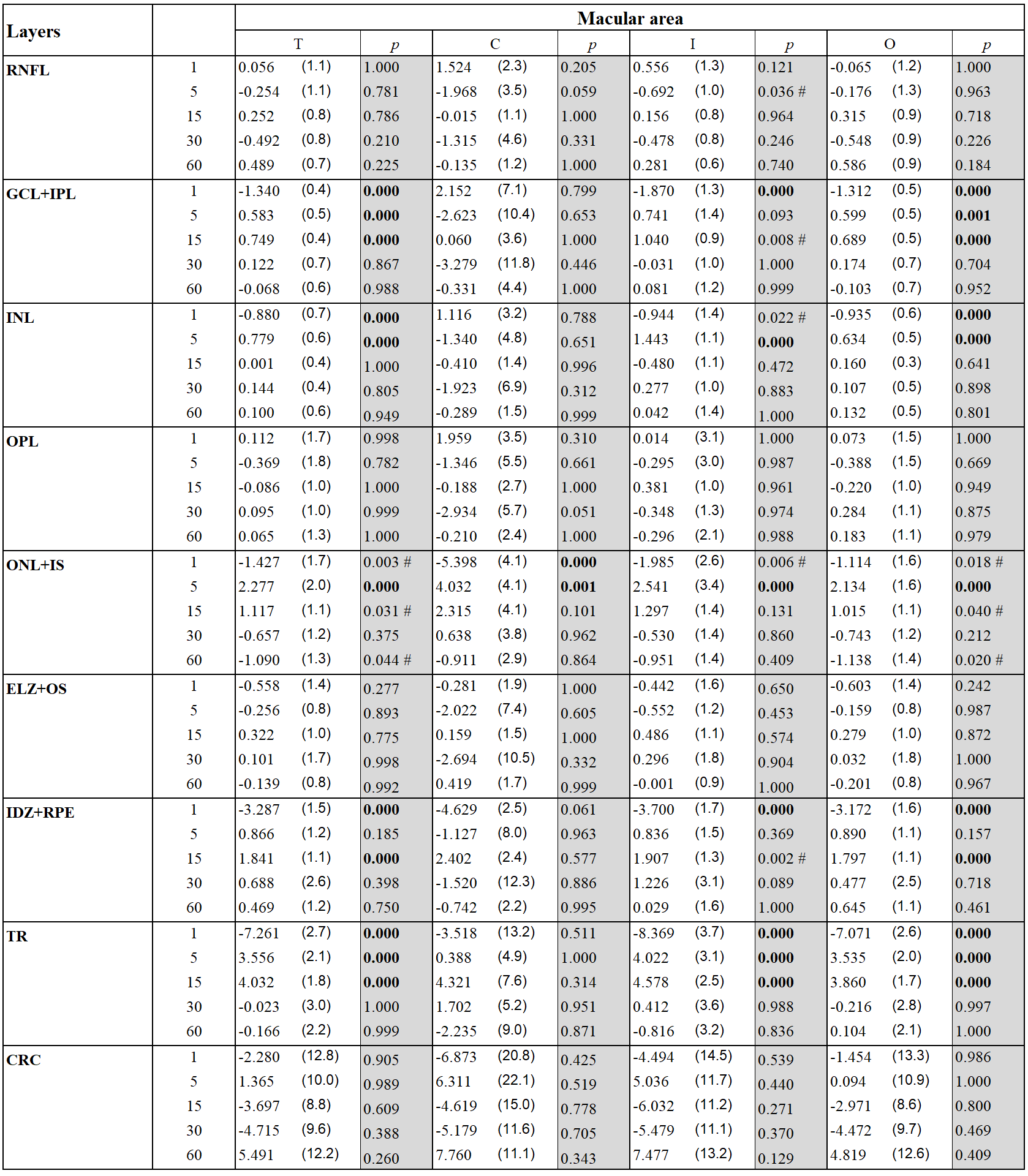

Supplement: S4 Table — The p values indicate the results of the post hoc Dunnett test in the case of significant ANOVA test. (TIF) [file pone.0268770.s004.tif]

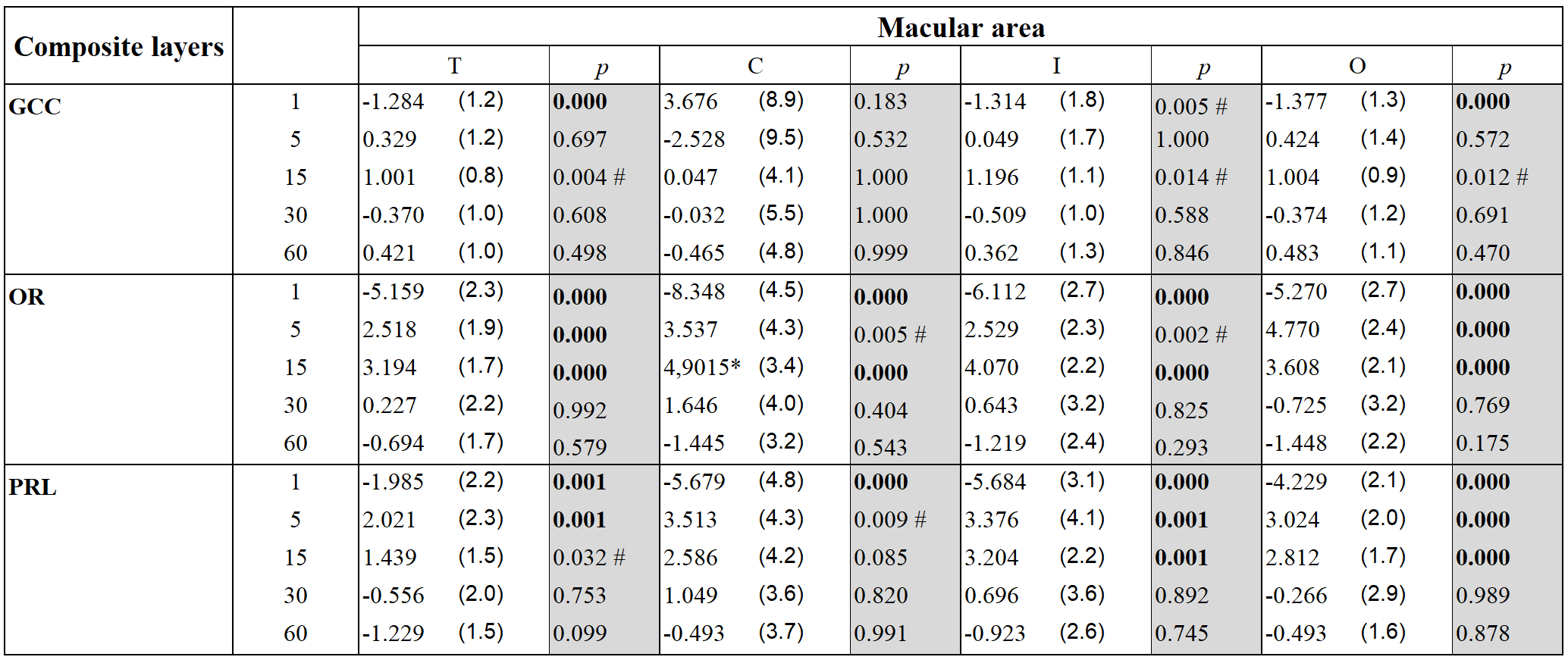

Supplement: S5 Table — The composite layers are the following: ganglion cell complex (GCC, RNFL+GCL+IPL), outer retina (OR, OPL+ONL+IS+ELZ+IS+IDZ+RPE) and photoreceptor layer (PRL, ONL+IS+ELZ+OS). The p values indicate the results of the post hoc Dunnett test in the case of significant ANOVA test. (For the abbreviations see Fig 2). (TIF) [file pone.0268770.s005.tif]
