# Supplementary material for: The assessment of acute chorioretinal changes due to intensive physical exercise in young adults
Source: PLoS One. 2022 May 25;17(5):e0268770. doi: 10.1371/journal.pone.0268770 (PMC9132279; doi:10.1371/journal.pone.0268770)
Supplement: Attachment — Submitted filename: Szalai_Response_to_Reviewers_R1.docx [file pone.0268770.s006.docx]

*We would like to thank both reviewers for their insightful comments. Please find below our detailed comments for all points raised in the review.*

Reviewer #1: First, i would like to congratulate the authors for their amazing work on investigating the correlation between chorioretinal thickness changes following intensive physical exercise.

*We would like to thank Reviewer #1 for his positive comment.*

Although the authors have fully and thoroughly highlighted the limitations to their research in the Discussion section, there are some concerns that should be addressed before this manuscript can be deemed significant for publication.

1- Using Pearson's correlation to examine the changes in chorioretinal thickness is a good choice given the lack of normality in the recorded observations. However, i think it would be better to conduct multiple linear regression models instead. This will help us determine if the change in chorioretinal thickness is really a reflection of change in physical activity that is not confounded by other baseline variables. The results of this analysis will not be definite as well, since certain confounders were not assessed during the recruitment period (i.e., axial length, IOP measurement), and this has to still be mentioned in the limitations section. I would suggest using time-dependent dependent variables. For example, the dependent variables would be: choroidal thickness at baseline; choroidal thickness change (from baseline to 5 minutes); choroidal thickness change (from 5 mins to 15 mins) and so on. This would give a better comprehension of the actual change in such measurement during each time interval.

*We agree with Reviewer #1 on the use of multiple linear regression and we indeed applied this methodology in our calculations. (See Page 10 Line 199: “Pearson correlation was calculated to assess the correlation of retinal thickness changes and multiple linear regression models with stepwise method were executed in order to assess which layers are changing together.”) As no results for confounders were found, we did not include anything in the text. To correct this, we now added a sentence in the results section referring to this. (See Page 16 Line 296: “**Multiple linear regression did not reveal any significant confounders.”) In the limitations section we are mentioning the lack of axial length and IOP measurements as a potential limiting factor of our study. (See Page 22 Line 424)*

*We also agree on the use of time-dependent dependent variables between adjacent time points. We did consider this at the time of statistical design but decided to use the baseline measurements for comparisons, i.e. baseline to 1 minute, baseline to 5 minutes and so forth, in order to assess relative change due to physical exercise. This also enables us to show thickness values of the retinal layers at different time points (and not the change values) on our figures that allows for a more intuitive perception and interpretation of our data. For the above reasons, we would remain with our original methodology.*

2- Did you put a limitation of participants' age during recruitment? (for example, >18 years of age)

If yes, then it needs to be mentioned in the methods section. And, if not, then based on what did you categorize them as young age?

*We would like to thank Reviewer #1 for this thoughtful comment. We included participants* *between 18-35 years of age, which we included in the Methods section. (See Page 5 Line 101)*

3- I would recommend adding a flow chart highlighting the recruitment process and the number of individuals included/excluded with reasons.

*We fully agree with Reviewer #1 on the need of transparency in terms of the recruitment process. We now included Fig. 1. highlighting this. (See Page 6 Line 122)*

4- Please replace reference 29 with a more updated/recent one.

*We now included a more recent Reference to Lunn WR et al.*

5- Please improve the quality of the figures provided.

*The figures are now saved in full resolution.*

6- In the introduction section: "The acute changes induced..............blood supply." Please add references that support each of the claims provided.

*We thank Reviewer #1 for this important comment and now included references to these sentences.*

7- In the conclusion section, both in the main text and in the abstract, please restrict your conclusions to the young age population.

*We revised the conclusion of the abstract and the manuscript text to highlight this. (See Page 3 Line 51 and Page 22 Line 441)*

8- Please remove the company name of the SD-OCT device from the abstract section.

*We removed the company name of the OCT device from the abstract.*

9- The English structure of the manuscript needs revision and editing by a native English speaker.

*We had our manuscript revised by a native English speaker to improve its language structure.*

*Again, we would like to express our gratitude for the important points raised by Reviewer #1 and for his efforts to improve our work.*

Reviewer #2: The authors describe a prospective study of the effect of exercise on the thickness of retino-choroidal layers in twenty-one eyes (four excluded due to poor data quality), in a population of young adults.

Retina-choroidal thickness was recorded in the 9 ETDRS regions, and was measured using OCT. Image analysis and segmentation was performed using custom software. Correlation was analysed between retina-choroidal thickness pre and post exercise. Other biometric parameters (height, weight, body mass index, blood pressure, heart rate, and refraction) were considered. The authors concluded that there was retinal thinning followed by thickening post exercise, no significant correlation between the retinal and choroidal changes were detected. Two mechanisms are discussed: circulatory auto-regulatory mechanisms and a mechanical effect of IOP on retinal thickness.

Comments:

The study was generally well designed, the statistical analysis was sound and the literature adequately reviewed. The omission of IOP measurement limits the interpretability of the findings particularly when the authors conclude that this may be one of the mechanisms that could explain the study findings and this factor is easily measured.

The article is technically sound, The statistical tests have been applied rigorously, the authors have made the data available for analysis and the manuscript is written in intelligible standard.

*We thank Reviewer #2 for his positive comments on our work. Indeed, the omission of IOP measurements is a limiting factor; however, we are dealing this in the most transparent fashion, encouraging the potential Readership of our paper to include this in future measurements.* *At the time of our study we had no technical possibility (i.e. an iCare device) that would have allowed the IOP measurements acutely after physical exercise, as our focus lied on the OCT assessments. We emphasized this point in the Discussion section of our manuscript. (See Page 22 Line 424: “however, we could not measure IOP after (and during) the physical load/strain as it would have been technically very demanding parallel with OCT imaging, the latter being of primary importance to us in this study.”)*

Minor recommendations:

Although the authors have used the standard conventions for abbreviations, the number of the abbreviations limits the readability, perhaps if a list of abbreviations could be provided in the end of the article.

*We agree with Reviewer #2; however, the format of PlosOne does not allow for the inclusion of an abbreviation list.*

*We would like to thank Reviewer #2 for his time and efforts to improve our manuscript.*
