# Supplementary material for: The assessment of acute chorioretinal changes due to intensive physical exercise in young adults
Source: PLoS One. 2022 May 25;17(5):e0268770. doi: 10.1371/journal.pone.0268770 (PMC9132279; doi:10.1371/journal.pone.0268770)
Supplement: Attachment — Submitted filename: Szalai_Response_to_Reviewers.docx [file pone.0268770.s007.docx]

*We would like to thank all the reviewers for their insightful and positive comments. Please find below our detailed comments for all points raised in the reviews.*

Reviewer #2:

The authors have addressed the comments. The analysis is technically sound and the conclusions are reasonable.

***Authors’ response:*** *We thank the Reviewer for his positive feedback.*

Reviewer #3

However there are some importante points that needs to be clarified in the study.
The authors indicate that 25 subjects were selected for the study. However, only 21 we qualified for the analysis and that is not clear in the abstract section.

***Authors’ response:*** *We thank the Reviewer for raising this important point that can indeed be confusing. Our Figure 1. shows the participant inclusion flowchart according to the STROBE guidelines. We included indeed 25 patients for the assessment of the demographic data; however, four subjects had missing OCT data and for this reason we excluded them from the OCT data assessment. Thus, we are reporting the data of 21 subjects, accordingly. We corrected this and made this clear throughout the entire text, including the abstract and also the Methods and Results section of the manuscript, in order to avoid confusion. (See Page 2 Line 27-28, Page 3 Line 48, Page 5 Line 101,103 and Page 10 Line 214)*

Very importante is that the sample (21 eyes) seems very small for investigating so many variables. The authors evaluated 12 thickness parameters at 4 macular regions. Therefore a total of 28 analysis were performed. Also such analysis were performed at baseline and a t 5 different time periods. Because of the large number of measurments and the small sample (21 eyes) the use of a p value of 0.5 seems inadequate to me. Although I understand that using Bonferroni’s correction would be too restrictive, the use of a p value of 0.05 with so many parameters and analysis would certainly lead to significant findings by chance alone. The authors should address that issue and use a more restrictive p value.

***Authors’ response:*** *We agree with Reviewer #3 that the large number of variables and the small number of subjects may skew the results which was highlighted in the weaknesses of the study in the discussion where the pilot experimental nature of our work has also been emphasized. In line with the advice of our professional statistician, our original concept was to present the data as they are due to the controversy in the international statistical community regarding the adjustments of significance levels. Nevertheless, to improve the presentation of our results we have modified the significance level to 0.001 (see Page 2 Line 35 and Page 10 Line 208) and restructured the entire text accordingly. We now present results between 0.05 and 0.001 as “missed significance” to help the Reader in the critical interpretation of our results; we decided to include these missed significant results in order to support the planning of future studies in the field regarding potential confounders.*

In table 3 the authors investigate the correlation of several thickness parameters with parameters age, SE, height, weight, BMI, BDP (our DBP, diastolic blood pressure) and Heart rate. I believe the use of person’s correlation would require that all parameters adhered to normality. The authors did not mention it by did the parameters adhere to normality? It seems unlikely to me.

***Authors’ response:*** *We completely agree with Reviewer #3 on the importance of normality testing prior to performing statistical assessments. Indeed, according to this we tested the normality of our data by Shapiro-Wilk test which can be found both on Page 10 Line 197 in the manuscript (“The Shapiro-Wilk test was used for normality testing. For normally distributed variables parametric tests were used and continuous data are reported as mean and standard deviation”).*

Also I believe evaluating the correlation between retina and choroid parameters with Age, SE, height, weight, BMI, DBP and HR is beyond the scope of the study and would require a larger number of subjects for obtaining representative results.

***Authors’ response:*** *Indeed, we conducted a relatively small study but we do believe that highlighting these correlations does make sense for the presentation of our results and to support the planning of future studies in the field. This weakness, among others, is now addressed in the Discussion section of the manuscript. (See Page 21 Line 423.)*

Data in Table 4 is also difficult to understand. Correlation between performance (PWR) and layer thickness changes ?? Did such parameters adhered to normality? What is the point of assessing such correlation? The large number of correlations assessed would easly explain obtaining some significant results (using a p level of 0.05). Since this is an exploratory investigation it would suffice to evaluate the measurements at diferente periods of time after exercise. I believe it is difficult to understand what is the meaning of the evaluation and its results.

***Authors’ response:*** *We thank the Reviewer for this valuable comment. We assessed the correlation between performance and the change of the layer thickness in order to assess whether the extent of physical activity influences the observed changes. The normal distribution of the data was checked also in this case (see our previous response). We are presenting our data in the revised manuscript based on the 21 subjects and using the modified p value of 0.001 as suggested by the Review. (See above.) (For the changes see Page 16, line 307-318)*

The authors compared the results of professionals and amateurs but the number of eyes in each group seems too small to make an adequate comparison.

***Authors’ response:*** *We are grateful to the Reviewer for raising this point. Our primary goal with this pilot study was the assessment of retinal changes in young adults. As the participants had different backgrounds regarding professional sports activities, we found it appealing to possibly compare or at least describe these two subgroups. We agree with the Reviewer that such a comparison would deliver questionable results and, therefore, already included it in the original Discussion of the study weaknesses (see Page 21 Line 422). We would prefer to leave it up to the Reader’s decision how they interpret these findings.*

Reviewer #4:

I want to congratulate the authors. It is a well written atricle with an interesting topic.

***Authors’ response:*** *We thank the Reviewer for his positive feedback.*

I have some issues 1-It is known that choroidal thickness and blood flow regulation may be altered in acute and chronic smokers, so it is better to indicate if the participants are acute or chronic smokers or not

***Authors’ response:*** *We would like to thank the Reviewer* for *this important comment. None of the subjects were smokers which we additionally included in the Results. (See Page 10 Line 215)*

2-Exclusion criteria will be defined more compherensive and detailed

***Authors’ response:*** *We have now all the exclusion criteria listed in the text (Page 6 Line 118)*

3-What did the authors do to standardize the intensity of the exercise between participants

***Authors’ response:*** *Thank you for raising this excellent point in the review. The way how intensity was standardized lies actually in the way we increased the loading itself, as originally described in the methods (Page 7 Line 131): each participant performed a stepwise incremental exercise trial until exhaustion (vita maxima) on* *a rowing ergometer. The intensity has been increased every 500 meters with the next load step required to be performed 10 seconds faster, until complete fatigue. The maximum power achieved in the last load step was considered in the evaluation. In order to further standardize the exercise intensity and make the values comparable among participants, for the assessments we used a calculated power-to-weight ratio (PWR, expressed in watt/kg). We believe this methodology enables a relatively unbiased comparison of the data.*

4-Were participants asked to alcohol or caffeinated drinks consumption or food or liquid ingestion before the exercise

***Authors’ response:*** *We are grateful to the Reviewer for this valuable comment.* *None of the subjects drank alcohol or caffeine at least 24 hours before the exercise. The meals were not prescribed; however, all subjects had had their regular breakfast at least one hour before the exercise. We added this information to the Methods section of the manuscript. (See Page 7 Line 128.)*
